# The limited role of RGS10 on NFκB and cytokine transcript levels in peritoneal macrophages under the influence of opioid analgesics

**DOI:** 10.1371/journal.pone.0343480

**Published:** 2026-09-23

**Authors:** Janna E. Jernigan Posey, Kruthika Dheeravath, Cassandra L. Cole, Noelle K. Neighbarger, Kelly B. Menees, Malú Gámez Tansey

**Affiliations:** 1 Center for Translational Research in Neurodegenerative Disease, College of Medicine, University of Florida, Gainesville, Florida, United States of America; 2 Department of Neuroscience, College of Medicine, University of Florida, Gainesville, Florida, United States of America; 3 McKnight Brain Institute, University of Florida, Gainesville, Florida, United States of America; 4 Department of Neurology, Indiana University School of Medicine, Indianapolis, Indiana, United States of America; 5 Stark Neuroscience Research Institute, Indianapolis, Indiana, United States of America; Macau University of Science and Technology, MACAO

## Abstract

Regulator of G-protein signaling 10 (RGS10) has been shown to regulate multiple inflammatory pathways relevant to disease pathogenesis. Of particular importance is the ability of RGS10 to negatively regulate the NFkB pathway, a prominent pro-inflammatory pathway implicated in multiple inflammatory disease phenotypes. However, the exact mechanism by which RGS10 regulates NFκB is unknown. Given that RGS10 translocates to the nucleus upon stimulation, we hypothesize that RGS10 may regulate NFκB at the transcript level. To determine whether RGS10 influences NFκB transcript levels, we stimulated peritoneal macrophages from RGS10 Knockout (KO) and B6 mice and collected cell lysate and conditioned media over a 24-hour period to assess transcript levels of NFκB and related pro-inflammatory cytokines as well as secreted cytokine levels. Here we found a limited and transient influence of RGS10 on transcript levels of NFκB subunits and NFκB-dependent cytokines. However, RGS10 displayed a more robust negative regulation of secreted protein levels of certain pro-inflammatory cytokines. Importantly, this study required the use of opioid analgesics prior to the collection of peritoneal macrophages and therefore reflects the role of RGS10 on pro-inflammatory transcript and protein levels when opioid analgesics are present. Overall, this study indicates that RGS10 may not serve as an important transcriptional regulator of NFκB related cytokine production under the influence of opioid analgesics. Further studies are warranted to understand the influence of opioid analgesics on RGS10 function and the mechanism by which RGS10 regulates NFκB activity.

## Introduction

Regulator of G-protein signaling 10 (RGS10) has been shown to regulate inflammatory responses that are protective in multiple chronic inflammatory disease models such as rheumatoid arthritis (RA), metabolic dysfunction, obesity, colitis, periodontitis, influenza infection, cancers, and even neurodegenerative disease models [1–9]. Mechanistically, RGS10 modulates multiple pathways that regulate the production of pro-inflammatory cytokines in myeloid cells including stromal interaction molecule 2 (STIM2) mediated calcium entry, glycolytic production of reactive oxygen species (ROS), and suppression of Nuclear Factor kappa-light-chain-enhancer of activated B cells (NFκB) [7,10,11]. Importantly, NFκB, a master transcriptional regulator of pro-inflammatory cytokines, is translocated to the nucleus upon inflammatory stimulation in myeloid cells allowing for the production of key inflammatory mediators such as Tumor necrosis factor (TNF), Interleukin 1-beta (IL-1B), Interleukin 6 (IL-6), Interleukin 12p40 (IL-12p40), and cyclooxegenase-2 (COX2) [12]. Dysregulated NFκB signaling has been identified as an important mediator of multiple chronic inflammatory diseases such as RA, Irritable bowel disease, type 1 diabetes, etc.[12]. Specifically, RGS10 deficiency enhances NFκB subunits protein levels upon inflammatory stimulus and also amplifies NFκB activity and pro-inflammatory cytokine secretion [4,7]. However, the mechanism by which RGS10 regulates NFκB levels is still unknown. Immunoprecipitation assays have further revealed that RGS10 does not have a direct protein-protein interaction with NFκB(10). Interestingly, both RGS10 and NFκB translocate to the nucleus upon inflammatory stimulation indicating that RGS10 may play a nuclear role in regulating the level and activity of NFκB(9, 12). Moreover, immunoprecipitation assays revealed that RGS10 does interact with DNA binding proteins indicating that it may play a role in transcription [10].

Therefore, we hypothesized that RGS10 may regulate NFκB transcript levels. To test this, we analyzed NFκB and pro-inflammatory transcripts of lipopolysaccharide (LPS) stimulated wild type C57B6/J (B6) and RGS10 Knock out (KO) peritoneal macrophages (pMacs) at 1,2,4,8, and 24h. Importantly, animals were given opioid analgesics prior to pMac collection. Under these conditions, we found that RGS10 regulates the transcription of NFκB subunits and NFκB-dependent cytokines in a diverse and limited capacity. Specifically, we demonstrate that RGS10 does not regulate the kinetics of NFκB subunit transcription or IL-1B cytokine transcript levels. However, we do see that RGS10 deficiency briefly results in higher levels of p65 transcript levels shortly after initial stimulation. Moreover, we found that RGS10 KO pMacs produced significantly less TNF transcript than their B6 counterparts and this difference was driven by male mice. We also found no difference in secreted protein levels of TNF, IL-1β, or IL-10 but did see elevated levels of IL-6, IL-12, and KCGRO in LPS stimulated RGS10 KO pMacs. In summary, we demonstrate a limited and transient regulation of differential NFκB pathway component transcripts by RGS10. These data indicate that RGS10 may not serve as a major regulator of NFκB related cytokine transcript production under the influence of opioid analgesics. Moreover, in comparison to previous studies our data suggests that myeloid cytokine secretion regulated by RGS10 may be altered by the influence of opioid analgesics. Further studies are warranted to understand the influence of opioid analgesics on RGS10 function and the mechanism by which RGS10 regulates NFκB activity.

## Materials and methods

Animals: Mice were housed in the McKnight Brain Institute vivarium at the University of Florida and maintained on a 12:12 light–dark cycle with *ad libitum* access to water and standard rodent diet chow. All animal procedures were approved by the Institutional Animal Care and Use Committee and followed the Guide for the Care and Use of Laboratory Animals from the National Institutes of Health at the University of Florida. Generation of the RGS10 Knock out (KO) line onto a C57B6/J background has been previously described [7]. 4- to 5-month-old male and female C57B6/J and RGS10 KO mice (n = 6/sex/group) were given 50 µL of sustained-release Buprenorphine (ZooPharm, Swedesboro, NJ) subcutaneously 30 minutes prior to intraperitoneal injection of 1mL of 3% thioglycolate. Three days later, animals were sacrificed via cervical dislocation for peritoneal macrophage collection.

Peritoneal macrophages: Peritoneal macrophage(pMacs) collection occurred as previously published [13]. Specifically, Pmacs collection occurred in a laminar flow hood. After cervical dislocation and spraying down the animals with 70% ethanol, 10mL of cold RMPI (ThermoFisher Scientific, 11875093) were injected into the peritoneum. Peritoneal lavage fluid was collected, passed through 70 uM filters prewet with 5mL of HBSS-/- (Gibco, 14175103, Grand Island, NY) into 50mL conical tubes. Filters were rinsed with 5mLs of HBSS-/- (Gibco, 14175103, Grand Island, NY) twice and centrifuged at 400xg for 5 minutes at 4°C. If blood cells were present in the sample, cells were treated with 1mL of ammonium-chloride-potassium lysis buffer for 1 min and then pelleted at 400xg for 5 minutes at 4°C. Cells were taken forward and processed in the biosafety hood using sterile technique. Supernatant was aspirated off cells, and cells resuspended in 3mL of warm plating media (RPMI, 10% fetal bovine serum, and 1% penicillin/streptomycin). 10 µL of cells were taken forward for counting using a 1:1 diluted trypan blue (1/4th trypan blue in sterile 1XPBS) stain and the CountessTM. Cells were plated at 1X10^6 density in 2 mL of culture media in a 12 well plate. Cells were placed in the incubator at 37° C and 5% CO2 to rest for 2 hours. After 2 hours, cells were washed with sterile DPBS (Gibco, 14190235, Grand Island, NY) to remove non-adherent cells and new pre-warmed culture media (RPMI supplemented with 10% FBS and 1x Pen-Strep) was added. Cells were treated with either vehicle or 100ng/mL LPS for 1,2,4,8, or 24 hs. Media was collected and flash frozen in liquid nitrogen. Cells were lysed with 350 µL Β – ME and RLT lysis buffer (20 µL of Β – ME per 1mL RTL buffer) and mechanically homogenized by scrapping each well with a cell scraper.

RNA extraction: Bench areas were cleaned with RNase Zap prior to RNA extraction. Lysed pMac cells were processed for RNA extraction via RNeasy Mini Kit (Qiagen, 74104, Hilden, Germany) according to manufacturer’s protocol. Briefly, lysed solution was transferred to Qiashredder tubes for complete homogenization. Qiashredder flow-though was stored in −80°C until sample batches were collected. Samples were thawed and mixed with 350 µL of 70% ethanol and transferred to RNAeasy spin columns. Samples were centrifuged for 15 seconds at 10,000rmp(9,391xg) at room temperature. Flow-through was discarded and nucleic acids trapped in the RNAeasy membrane were washed with 700 µL of RW1 buffer and 500 µL of RPE buffer twice, centrifuging for 15 seconds at 10,000rmp at room temperature and discarding flow-through after each wash. Samples were then centrifuged for 2 minutes at full speed to dry before eluting samples with 30 µL of RNAse free water in a 1-minute spin at 10,000 rpm. RNA concentrations were determined using Denovix spectrophotometer and samples were stored at −80°C prior to cDNA synthesis.

cDNA synthesis: DNA was removed from RNA samples prior to cDNA synthesis by diluting RNA samples with nuclease free water and 3.68µl of DNAse master mix (3.36µl of 25mM Mgcl2 + 0.32µl of 1/5 DNAse 1) reaching a total volume of 10µl per sample. Samples were then run through the thermocycler at 37° C for 30 minutes followed by 75° C for 10 minutes and a 4° C hold. 0.4ug of cDNA for each sample was synthesized from RNA using the High-Capacity cDNA Reverse Transcription Kit (Thermo Fisher Scientific, 4368814, Foster City, CA) according to manufacturer’s instructions. Briefly, 10µl of reverse transcription master mix, which was comprised of 2µl of 10XRT Buffer, 0.8 25XdNT mix (100mMm, 2µl of 10XRT random Primers, 1µl of Rnase Inhibitor, 1µl of Reverse Transcriptase, and 3.2µl of Nuclease-Free water per sample, was added to each DNA-free sample. Samples were vortexed and spun down and run in the thermocycler at 25° C for 10 minutes followed by 37° C for 2 hours then 85° C for 5 minutes and a 4° C hold. cDNA samples were then diluted to the final concentration with nuclease-free water and stored at −20 ° C.

Real Time Quantitative PCR: cDNA was combined with PowerTrack™ SYBR Green Master Mix for qPCR (Thermo Fisher Scientific, A46112) and specific Integrated DNA technologies (IDT) Forward and Reverse primers at 10ng/well reactions in a 384 well plate in triplicate for Real Time Quantitative PCR analysis in comparative CT run on the Quant Studio 5 at a volume of 20µl/well. Primer sequences were as follows: mouse NFκB subunit p65 forward: GGA TCC AGT GTG TGA AGA AG and reverse: CTC CTC TAT AGG AAC GTG AAA G, mouse NFκB subunit p50 forward: GGG ACA GTG TCT TAC ACT TAG and reverse: GTC ATC AGA GAT CAA ACC AGA, mouse RGS10 forward: TTG GCT AGC GTG TGA AGA TTT C and reverse: TGG CCT TTT CCT GCA TCT G, mouse TNF forward: CTG AGG TCA ATC TGC CCA AGT AC and reverse: CTT CAC AGA GCA ATG ACT CCA AAG, mouse IL-1B forward: CAA CCA ACA AGT GAT ATT CTC CAT G and reverse: GAT CCA CAC TCT CCA GCT GCA. All QPCR samples were normalized to their own Beta- Actin CT and then to the WT B6 saline group. Samples with an average triplicate variance of ≥ 0.3, had outlying triplicate removed and if samples average remaining variance still exceeded 0.3 the sample was excluded from analysis.

Mesoscale Discovery Multiplexed Immunoassays: Protein concentrations of TNF, IL-6, IL-1β, KC/GRO, IL-4, IL-10, IFN-Y, IL-5, IL-2, and IL-12p70 in conditioned media were analyzed (MesoScale Discovery, K15048D-2, Rockville, Maryland) using multiplexed immunoassays on the MesoScale Discovery (MSD) platform. Samples were plated in duplicate at a 1:1 dilution with MSD diluent 41. Samples were processed according to the manufacturer’s instructions as previously described [13]. Plates were analyzed on the MSD Quickplex machine and MSD software (Discovery Workbench Version 4.0). Cytokines with values below the lower limit of detection (LLOD) were not quantified. Cytokine protein levels were normalized to total RNA yield to accommodate for any well-to-well variability in cell numbers.

Statistics: Analyses were performed with Graph-Pad Prism 10.6. Group differences were analyzed using Ordinary three-way ANOVA corrected for multiple comparisons with Tukey post hoc test. Differences between groups across time were analyzed using one-way ordinary ANOVA corrected for multiple comparisons with Tukey’s post hoc analysis. Statistically different samples do not share the same letter. p values ≤ 0.05 were considered statistically significant.

## Results

**Fig 1:**
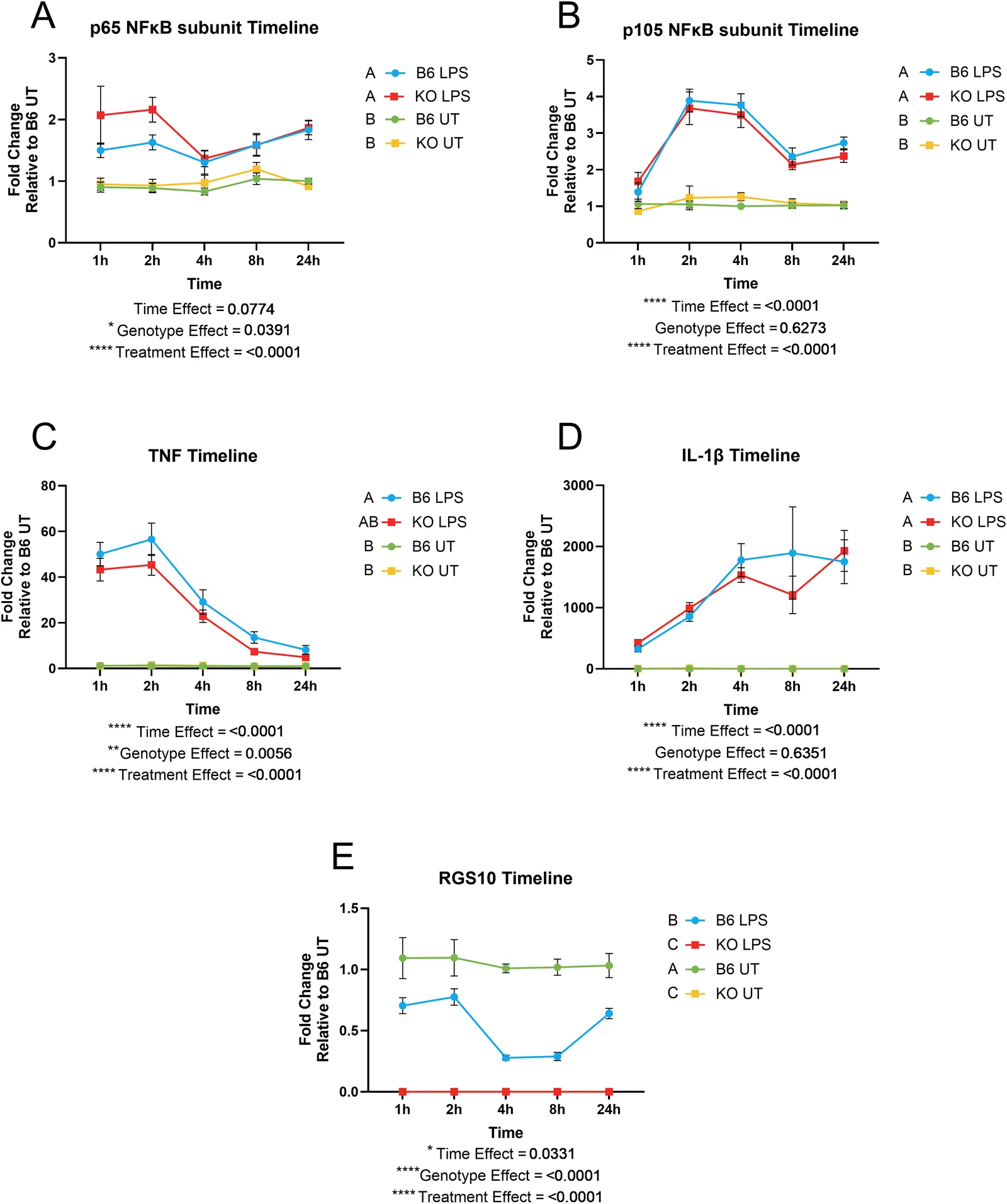
RGS10 has a limited influence on NFκB subunits and NFκB-dependent transcript levels. N = 12/group. The red line represents RGS10 KO pMacs treated with LPS, blue line represents B6 pMacs treated with LPS, yellow line represents RGS10 KO pMacs treated with vehicle, and the green line represents B6 pMacs treated with vehicle. A) Fold change of p65 NFκB transcripts relative to control over 24h. B) Fold change of p50 NFκB transcripts relative to control over 24h C) Fold change of TNF transcripts relative to control over 24h. D) Fold change of IL-1β transcripts relative to control over 24h. E) Fold change of RGS10 transcripts relative to control over 24h. Main effects p values are present below each graph. Statistically different samples do not share the same letter.

To determine whether the RGS10 mediated NFκB-dependent transcript levels, we stimulated RGS10 KO and B6 peritoneal macrophages and collected cell lysate over a 24-hour period to  assess transcript levels of NFκB and related pro-inflammatory cytokines. If the hypothesis that RGS10 negatively regulates NFκB transcript levels is correct, we would expect to see that RGS10 deficiency enhances NFκB-dependent pro-inflammatory gene transcription. Here, we find that RGS10 does not differentially regulate the kinetics of NFκB subunit transcription when analyzed over the course of 24 hours, nor did we find an enhancement in the transcription of NFκB-dependent genes such as TNF and IL-1B (Fig 1, S2-S4 Figs). However, we did find that RGS10 deficiency results in higher levels of the p65 NFκB subunit transcript at 2 hours post LPS stimulation (S1D Fig), indicating a potential time-sensitive role for the regulation of the p65 NFκB subunit by RGS10. Importantly, we demonstrate that RGS10 transcript levels are reduced upon stimulation and recover over time, which has been previously reported by multiple studies (Fig 1B, S5 Fig) [9,14]. Interestingly, we found a genotype effect of TNF transcripts indicating that RGS10 KO peritoneal macrophages (pMacs) were producing less TNF than their B6 counterparts (Fig 1C).

**Fig 2:**
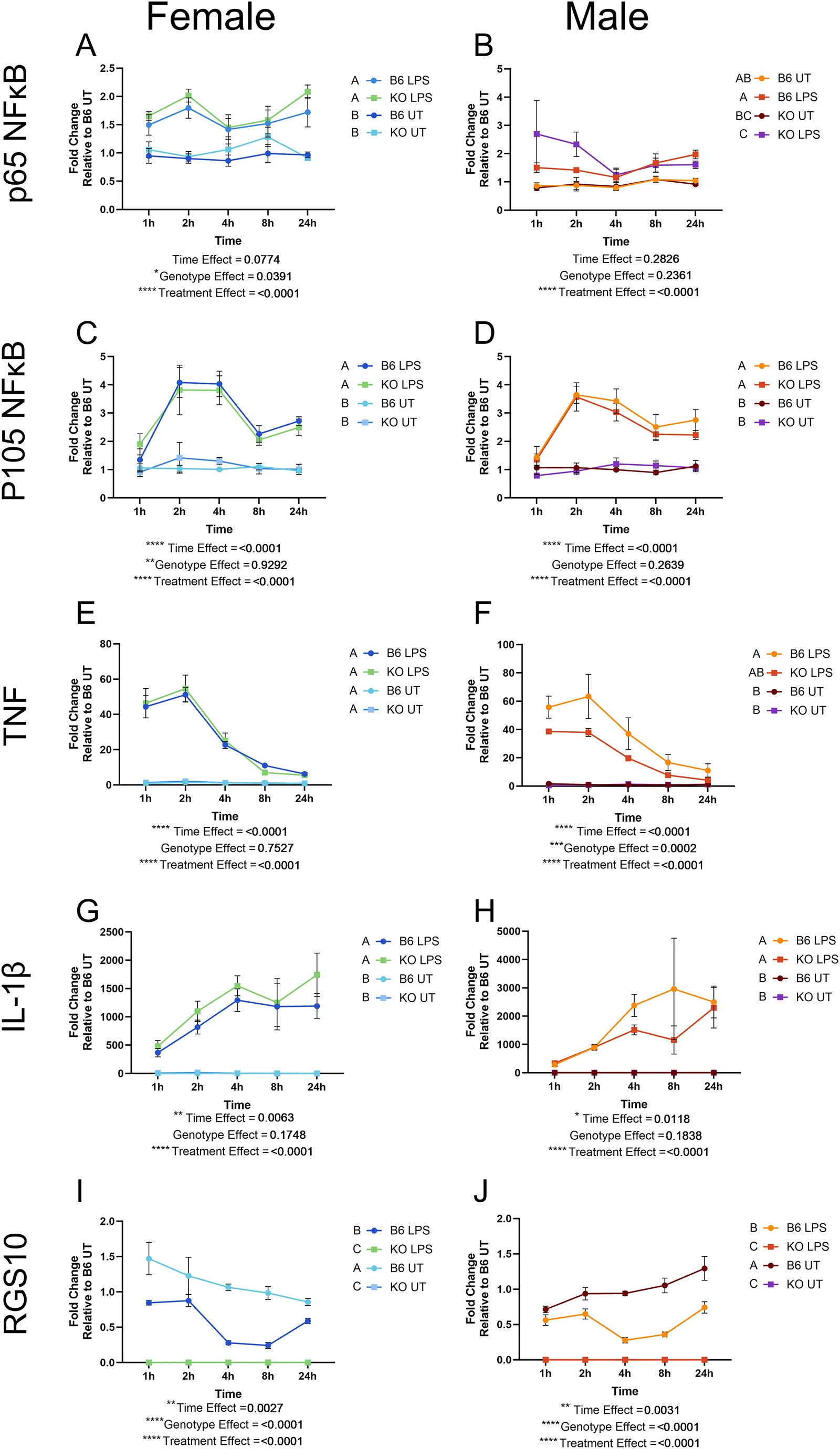
Transcription of the NFκB subunits and pro-inflammatory cytokines is similar across both sexes. N = 6/sex/group. Fold change of p65 NFκB transcripts relative to control over 24h in females (A) and males (B)**.** Fold change of p50 NFκB transcripts relative to control over 24h in females (C) and males (D). Fold change of TNF transcripts relative to control over 24h in males (E) and females (f). Fold change of IL-1βtranscripts relative to control over 24h in males (G) and females (H). Fold change of RGS10 transcripts relative to control over 24h in males (I) and females (J). Main effect p values are present below each graph. Statistically different samples do not share the same letter.

To determine whether any of our results were sex-specific, we analyzed our data in males and females separately. Here we found that the genotype effect on TNF is only present in male RGS10 KO pMacs and not females (Fig 2C, 2G). Otherwise, male and female pMacs produced similar transcriptional inflammatory responses to LPS. Interestingly, early transcription of the p65 subunit of NFκB was significantly enhanced with RGS10 deficiency in females but not males (S1E-S1F Fig).

**Fig 3:**
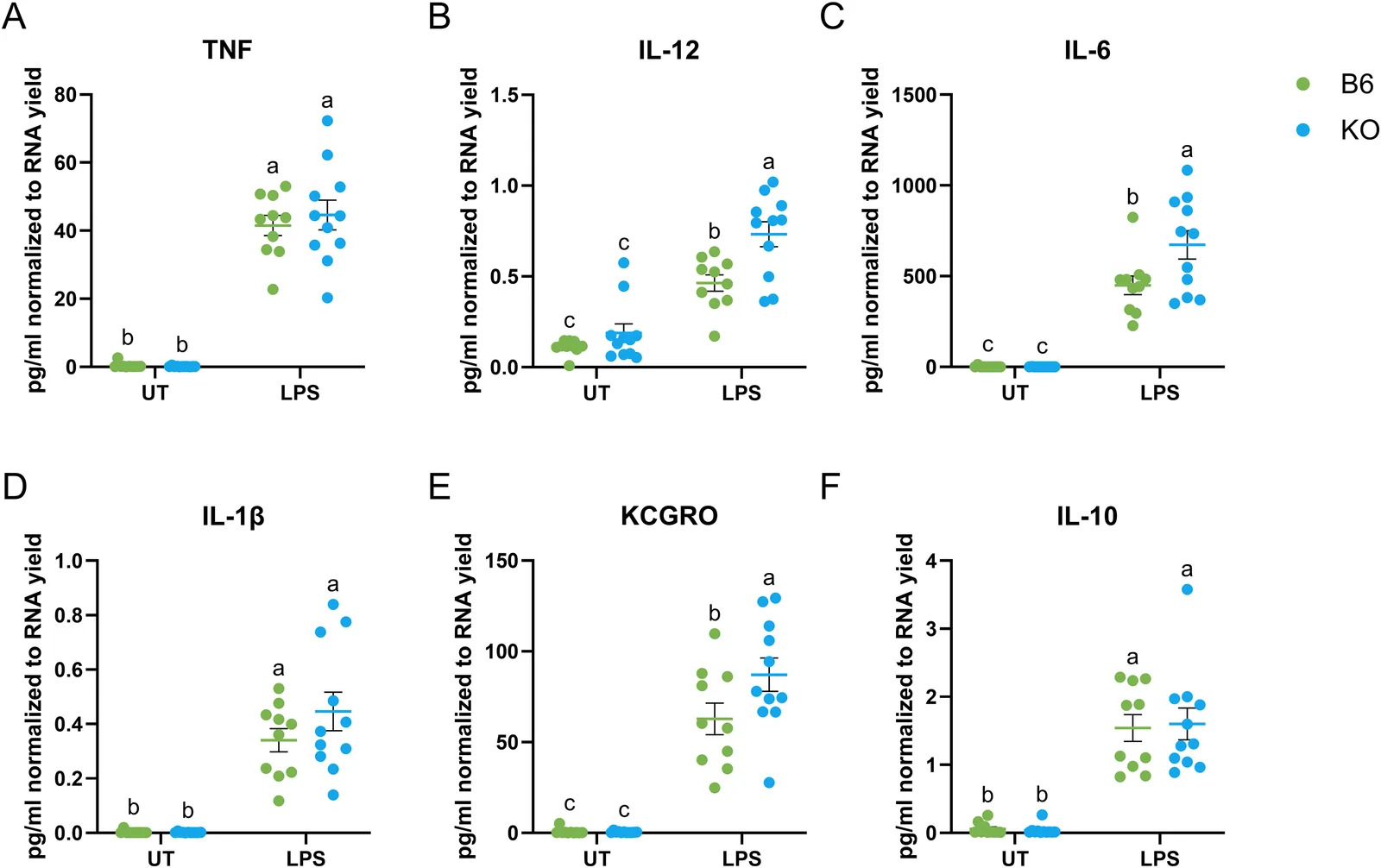
RGS10 regulates the secretion of pro-inflammatory cytokines in peritoneal macrophages. Level of pro-inflammatory cytokines from male and female pMacs N = 12/group. Cytokines with values below the lower limit of detection (LLOD) were not quantified. Green dots are B6 pMacs and blue dots are RGS10 KO pMacs. A) pg/mL normalized to RNA yield of TNF protein levels 24h post stimulation. B) pg/mL normalized to RNA yield of IL-12 protein levels 24h post stimulation. C) pg/mL normalized to RNA yield of IL-6 protein levels 24h post stimulation. D) pg/mL normalized to RNA yield of IL-1B protein levels 24h post stimulation. E) pg/mL normalized to RNA yield of KCGRO protein levels 24h post stimulation. F) pg/mL normalized to RNA yield of IL-10 protein levels 24h post stimulation. Statistically different samples do not share the same letter.

It was surprising that our data did not demonstrate any enhancement in TNF or IL-1β transcripts levels as previous literature reported exacerbated TNF and IL-1β protein levels with RGS10 KO in pMacs [15]. Therefore, we examined the level of secreted pro-inflammatory cytokines from the conditioned media. Again, we found that TNF and IL-1β levels did not change with RGS10 deficiency, nor did levels of IL-10 (Fig 3A,3D,3F). Instead, we found that RGS10 deficiency exacerbated the levels of IL-6, KCGRO, and IL-12 (Fig 3B, 3C, 3E).

## Discussion

Overall, our data demonstrates that RGS10 has a limited influence over the levels of NFκB subunits and NFκB-dependent cytokines transcripts in peritoneal macrophages. Moreover, this effect is time sensitive and displays multiple sex biases. The brief, non-sustained increase in p65 NFκB subunit transcript levels with RGS10 deficiency can likely be explained by the downregulation of RGS10 transcript post LPS stimulation, rending a lack of RGS10 in which to regulate p65 NFκB transcript levels with past the initial stimulation (~within 2 hours of stimulation). In support of this idea, RGS10 protein levels have also been observed to decrease with LPS stimulation in other myeloid populations over the course of 24–48 hours prior to returning to baseline levels [9,14].

Moreover, our data reveals novel cytokine transcription and secretion patterns mediated by RGS10. Specifically, we observe, in contrast to previous studies, that RGS10 deficiency did not impact transcript levels of of key NFκB-dependent cytokine, IL-1β, and even resulted in the reduction of TNF transcript in a sex-dependent manner. Interestingly, IL-6, KCGRO, and IL-12 cytokine secretion was exacerbated by RGS10 deficiency instead, capturing a differential inflammatory profile that is regulated by RGS10. Importantly, this differential mediation of inflammation may be due to the use of buprenorphine in our study that previous studies were not stipulated to use [15]. Buprenorphine and other opioids such as morphine have been shown to decrease pro-inflammatory cytokines such as IL-1B and TNF in macrophages, among other functional changes [16–18]. Moreover, buprenorphine is a partial mu opioid receptor agonist, and the mu opioid receptor, which is expressed in myeloid cells among others, is an inhibitory GPCR [19]. RGS10 was canonically identified as GTPase-activating protein with high-affinity for inhibitory GPCRs [20]. Therefore, we speculate that the regulation of pro-inflammatory responses by RGS10 may be significantly influenced by the use of opioids. Follow-up studies are necessary to clarify the influence of buprenorphine and other opioids on RGS10-mediated myeloid cell activation. Moreover, this study is limited to the assessment of transcript levels and did not directly assess possible mechanisms by which RGS10 may influence transcript levels. Future studies to assess the impact of RGS10 on NFκB promoter activity and possible chromatin binding capacity should be explored to address this gap. Consequently, our study reveals that RGS10 transiently regulates the transcript levels of NFκB pathway components in a limited manner and that RGS10 may mediate alternative inflammatory responses under the influence of opioid analgesics.

## Supporting information

S1 Fig Supplemental Figure 1: RGS10 suppresses p65 NFKB subunit transcription during initial stimulation response. Fold change of the transcripts for the p65 subunit of NFKB relative to control at 1h post stimulation in both sexes (A), females (B), and males (C). Fold change of the transcripts for the p65 subunit of NFKB relative to control at 2h post stimulation in both sexes (D), females (E), and males (F). Fold change of the transcripts for the p65 subunit of NFKB relative to control at 4h post stimulation in both sexes (G), females (H), and males (I). Fold change of the transcripts for the p65 subunit of NFKB relative to control at 8h post stimulation in both sexes (J), females (K), and males (L). Fold change of the transcripts for the p65 subunit of NFKB relative to control at 24h post stimulation in both sexes (M), females (N), and males (O). Samples that are statistically different do not share the same letter. Group differences were analyzed using Ordinary two-way Anova corrected for multiple comparisons with Tukey post hoc test. p values ≤ 0.05 were considered statistically significant.
(TIF)

S2 Fig Supplemental Figure 2: RGS10 does not modulate p105 NFKB subunit transcription. Fold change of the transcripts for the p105 subunit of NFKB relative to control at 1h post stimulation in both sexes (A), females (B), and males (C). Fold change of the transcripts for the p105 subunit of NFKB relative to control at 2h post stimulation in both sexes (D), females (E), and males (F). Fold change of the transcripts for the p105 subunit of NFKB relative to control at 4h post stimulation in both sexes (G), females (H), and males (I). Fold change of the transcripts for the p105 subunit of NFKB relative to control at 8h post stimulation in both sexes (J), females (K), and males (L). Fold change of the transcripts for the p105 subunit of NFKB relative to control at 24h post stimulation in both sexes (M), females (N), and males (O). Samples that are statistically different do not share the same letter. Group differences were analyzed using Ordinary two-way Anova corrected for multiple comparisons with Tukey post hoc test. p values ≤ 0.05 were considered statistically significant.
(TIF)

S3 Fig Supplemental Figure 3: RGS10 deficiency reduces the transcription of TNF over the course of 24h primarily in males. Fold change of the transcripts for TNF relative to control at 1h post stimulation in both sexes (A), females (B), and males (C). Fold change of the transcripts for TNF relative to control at 2h post stimulation in both sexes (D), females (E), and males (F). Fold change of the transcripts for TNF relative to control at 4h post stimulation in both sexes (G), females (H), and males (I). Fold change of the transcripts for TNF relative to control at 8h post stimulation in both sexes (J), females (K), and males (L). Fold change of the transcripts for TNF relative to control at 24h post stimulation in both sexes (M), females (N), and males (O). Samples that are statistically different do not share the same letter. Group differences were analyzed using Ordinary two-way Anova corrected for multiple comparisons with Tukey post hoc test. p values ≤ 0.05 were considered statistically significant.
(TIF)

S4 Fig Supplemental Figure 4: RGS10 does not modulate IL-1B transcription. Fold change of the transcripts for IL-1B relative to control at 1h post stimulation in both sexes (A), females (B), and males (C). Fold change of the transcripts for IL-1B relative to control at 2h post stimulation in both sexes (D), females (E), and males (F). Fold change of the transcripts for IL-1B relative to control at 4h post stimulation in both sexes (G), females (H), and males (I). Fold change of the transcripts for IL-1B relative to control at 8h post stimulation in both sexes (J), females (K), and males (L). Fold change of the transcripts for IL-1B relative to control at 24h post stimulation in both sexes (M), females (N), and males (O). Samples that are statistically different do not share the same letter. Group differences were analyzed using Ordinary two-way Anova corrected for multiple comparisons with Tukey post hoc test. p values ≤ 0.05 were considered statistically significant.
(TIF)

S5 Fig Supplemental Figure 5: LPS stimulation reduces RGS10 transcription levels over the course of 24h. Fold change of the transcripts for RGS10 relative to control at 1h post stimulation in both sexes (A), females (B), and males (C). Fold change of the transcripts for RGS10 relative to control at 2h post stimulation in both sexes (D), females (E), and males (F). Fold change of the transcripts for RGS10 relative to control at 4h post stimulation in both sexes (G), females (H), and males (I). Fold change of the transcripts for RGS10 relative to control at 8h post stimulation in both sexes (J), females (K), and males (L). Fold change of the transcripts for RGS10 relative to control at 24h post stimulation in both sexes (M), females (N), and males (O). Samples that are statistically different do not share the same letter. Group differences were analyzed using Ordinary two-way Anova corrected for multiple comparisons with Tukey post hoc test. p values ≤ 0.05 were considered statistically significant.
(TIF)

## Abbreviations

β – ME: Beta mercaptoethanol
cDNA: complementary DNA
Cox2: Cyclooxygenase 2
DNA: Deoxyribonucleic Acid
DPBS: Dulbecco’s Phosphate Buffered Saline
GPCR: G Protein Coupled Receptor
IL-12: Interleukin 12
IL-12p40: Interleukin 12p40
IL-1β: Interleukin 1β
KCGRO: Keratinocyte chemoattractant/Growth-regulated oncogene
KO: Knock Out
LLOD: Lower limit of detection
LPS: Lipopolysaccharide
MSD: Mesoscale Discovery
NFκB: Nuclear Factor kappa-light-chain-enhancer of activated B cells
pMac: Peritoneal macrophages
QPCR: Quantitative real time polymerase chain reaction
RA: Rheumatoid Arthritis
RGS10: Regulator of G Protein Signaling 10
RNA: Ribonucleic Acid
RPMI 1640: Roswell Park Memorial Institute 1640
STIM2: Stromal interaction molecule 2
TNF: Tumor Necrosis Factor
WT: Wild type
